# The Clinical Efficacy and Safety of Anti-Viral Agents for Non-Hospitalized Patients with COVID-19: A Systematic Review and Network Meta-Analysis of Randomized Controlled Trials

**DOI:** 10.3390/v14081706

**Published:** 2022-08-02

**Authors:** Chih-Cheng Lai, Ya-Hui Wang, Kuang-Hung Chen, Chao-Hsien Chen, Cheng-Yi Wang

**Affiliations:** 1 Division of Hospital Medicine, Department of Internal Medicine, Chi Mei Medical Center, Tainan 710, Taiwan; dtmed141@gmail.com; 2 Medical Research Center, Cardinal Tien Hospital and School of Medicine, College of Medicine, Fu Jen Catholic University, New Taipei City 231, Taiwan; yhwang531@gmail.com; 3 Department of Internal Medicine, National Taiwan University Hospital, College of Medicine, National Taiwan University, Taipei 100, Taiwan; jetcgh@gmail.com; 4 Division of Pulmonary, Department of Internal Medicine, MacKay Memorial Hospital, Taipei 104, Taiwan; 5 Department of Medicine, MacKey Medical College, New Taipei City 252, Taiwan; 6 Department of Internal Medicine, Cardinal Tien Hospital and School of Medicine, College of Medicine, Fu Jen Catholic University, New Taipei City 231, Taiwan

**Keywords:** COVID-19, nirmatrelvir plus ritonavir, remdesivir, molnupiravir

## Abstract

This network meta-analysis compared the clinical efficacy and safety of anti-viral agents for the prevention of disease progression among non-hospitalized patients with COVID-19. PubMed, Embase, Web of Science, Cochrane Library, ClinicalTrials.gov and the WHO International Clinical Trials Registry Platform were searched from their inception to 28 May 2022. Only randomized controlled trials (RCTs) that investigated the clinical efficacy of anti-viral agents for non-hospitalized patients with COVID-19 were included. Three RCTs involving 4241 patients were included. Overall, anti-viral agents were associated with a significantly lower risk of COVID-19 related hospitalization or death compared with the placebo (OR, 0.23; 95% CI: 0.06–0.96; *p* = 0.04). Compared with the placebo, patients receiving nirmatrelvir plus ritonavir had the lowest risk of hospitalization or death (OR, 0.12; 95% CI: 0.06–0.24), followed by remdesivir (OR, 0.13; 95% CI: 0.03–0.57) and then molnupiravir (OR, 0.67; 95% CI: 0.46–0.99). The rank probability for each treatment calculated using the P-score revealed that nirmatrelvir plus ritonavir was the best anti-viral treatment, followed by remdesivir and then molnupiravir. Finally, anti-viral agents were not associated with an increased risk of adverse events compared with the placebo. For non-hospitalized patients with COVID-19 who are at risk of disease progression, the currently recommended three anti-viral agents, nirmatrelvir plus ritonavir, molnupiravir and remdesivir, should continue to be recommended for the prevention of disease progression. Among them, oral nirmatrelvir plus ritonavir and intravenous remdesivir seem to be the better choice, followed by molnupiravir, as determined by this network meta-analysis. Additionally, these three anti-viral agents were shown to be as tolerable as the placebo in this clinical setting.

## 1. Introduction

Since the emergence of severe acute respiratory syndrome-coronavirus-2 (SARS-CoV-2) at the end of 2019, coronavirus disease 2019 (COVID-19) quickly presented a severe threat to public health [1]. As of 13 June 2022, there have been a total of 532,887,351 confirmed cases of COVID-19 and more than 6 million deaths [2]. Clinically, most patients with SARS-CoV-2 infection present as asymptomatic or with a mild illness requiring outpatient treatment, and only a small portion go on to develop severe or critical COVID-19 requiring hospitalization or intensive care unit management [3]. Therefore, how to prevent patients with SARS-CoV-2 infection from progressing to severe COVID-19 has become an important issue, especially for high-risk patients. The prevention of disease progression can reduce the morbidity and mortality of COVID-19 as well as the burden on the health care system.

To date, several therapeutic options have been developed and utilized to treat non-hospitalized adults with mild to moderate COVID-19 to try to prevent disease progression. This included anti-viral agents such as nirmatrelvir plus ritonavir, molnupiravir, and remdesivir, and neutralizing monoclonal antibodies, such as bamlanivimab, bamlanivimab/etesevimab, casirivimab/imdevimab, regdanvimab, sotrovimab and bebtelovimab [4,5,6,7,8,9,10,11]. Although many of these agents appeared promising in vitro and showed clinically preventive efficacy in initial studies [5,6,7,8,10,11], some neutralizing monoclonal antibodies have shown decreased activity against omicron variants of SARS-CoV-2 (including the omicron BA.2 subvariant) and are no longer recommended for this clinical entity. At present, only three antiviral agents are recommended for use in COVID-19 patients: nirmatrelvir plus ritonavir, molnupiravir and remdesivir [4,12,13]. However, a head-to-head comparison of these anti-viral agents is lacking. Therefore, we conducted this network meta-analysis to compare the clinical efficacy and safety of anti-viral agents for the prevention of disease progression among non-hospitalized patients with COVID-19.

## 2. Materials and Methods

### 2.1. Methods

This systematic review and network meta-analysis was conducted in accordance with the Preferred Reporting Items for Systematic Reviews and Meta-analyses (PRISMA) reporting guidelines [13]. The protocol of this study was registered at PROSPERO with a registration number of CRD42022335155.

### 2.2. Search Strategy

PubMed, Web of Science, Embase, Cochrane Library, ClinicalTrials.gov and the WHO international clinical trials registry platform were searched from their inception to 28 May 2022. We also manually searched for additional eligible articles from the reference lists of relevant articles. We used the following search terms: COVID-19, nirmatrelvir, molnupiravir, remdesivir, and randomized controlled study (RCT). Detailed search strategies are listed in Table S1.

### 2.3. Study Selection

Two investigators (CCL and CHC) independently screened the titles and abstracts of the studies collected using the aforementioned search strategies to identify and assess potentially eligible studies. Disagreements were resolved by a third investigator (CYW). Full-text copies of potentially relevant articles were obtained and reviewed for eligibility. Only RCTs that investigated the clinical efficacy of anti-SARS-CoV-2 therapy for non-hospitalized patients with COVID-19 were included. No limitations were imposed regarding language, age, sex, race or ethnicity, or duration of treatment. Studies were included if they met the following criteria: (i) included non-hospitalized adult patients with COVID-19; (ii) included treatment with molnupiravir, remdesivir or nirmatrelvir; (iii) compared with a placebo, standard of care or other anti-viral treatment; (iv) study design was an RCT, either blinded or open labelled; and (v) reported at least one of the following two outcomes: the risk of hospitalization or death. The following were excluded: (i) conference posters, case reports, case series, and observational studies; (ii) single-arm studies; (iii) studies that did not report the outcomes of interest; (iv) pharmacokinetic studies; and (v) nonhuman studies.

### 2.4. Data Extraction

The following information was extracted from the included studies by two investigators separately, and any disagreements were resolved by the third investigator: author name, year of publication, journal name, study title, inclusion criteria, exclusion criteria, primary end point and secondary end point. The following background information was extracted: number of patients, treatment regimen and the duration of treatment. The primary outcome was the risk of COVID-19-related hospitalization or death and the secondary outcome was the risk of adverse events (AEs).

### 2.5. Assessment of Risk of Bias

The quality of each included study was assessed using the revised Cochrane risk of bias tool [14]. Two investigators (YHW AND KHC) subjectively reviewed all the included studies and rated them “low risk”, “some concerns”, or “high risk” according to the bias in the following domains: randomization process, deviations from intended interventions, missing outcome data, measurement of the outcome and selection of the reported result. Disagreements were resolved through discussion and consensus with a third investigator (CHC).

### 2.6. Statistical Analysis

Odds ratios (ORs) were considered measures of effect size for categorical outcomes. Statistical significance was considered if the 95% confidence interval (95% CI) did not include 1 for ORs. A network meta-analysis with a random-effects model was performed for data synthesis that combined direct and indirect evidence across studies that evaluated multiple treatments. A frequentist approach was performed using the R package ‘netmeta’. The ranking of treatment that is an analogue of the surface under the cumulative ranking (SUCRA) was calculated using the P-score; a higher probability indicated better treatment [15]. Network graphs were also plotted for the primary outcome. The nodes represent treatment, and the edges represent the number of studies that provided results of direct comparison between two treatments. The size of the nodes was proportional to the number of patients included in the treatment, and the thickness of the edges was proportional to the number of included studies with direct evidence.

## 3. Results

### 3.1. Search Results and Characteristics of the Included Studies

**Figure 1:**
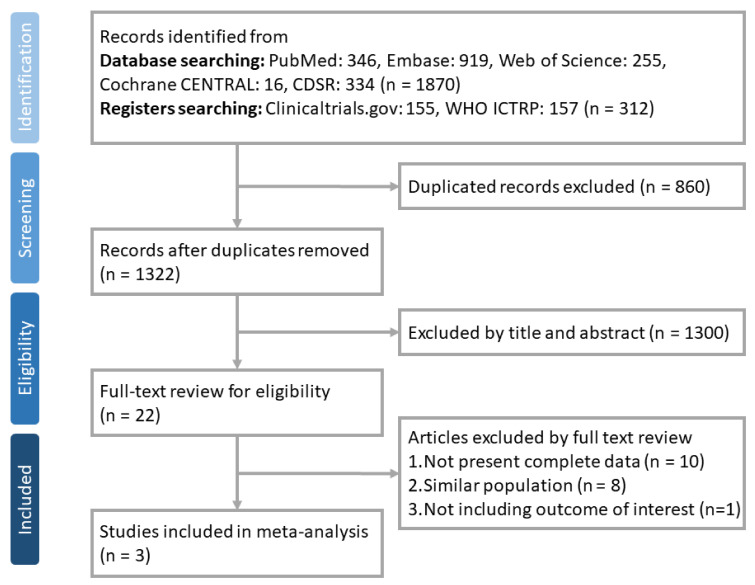
The flow diagram of the identification, inclusion, and exclusion of studies.

We initially identified 2182 studies. After excluding 860 duplicate articles, 1322 articles were screened, 1300 of which were excluded based on the title and abstract. The 22 remaining articles underwent a full-text review to assess their eligibility. Finally, a total of three RCTs [5,6,7] were identified that met the selection criteria. The algorithm for study selection is shown in Figure 1. Registered clinical trials not yet published are listed in Table S2. Table 1 summarizes the characteristics of the three included studies. All were double-blind placebo-control, multination, multicenter studies. The included patients were non-hospitalized, unvaccinated patients with COVID-19 who were at risk for progression to severe disease. The studied anti-viral agents included five days of oral nirmatrelvir plus ritonavir [5], five days of oral molnupiravir [6] and three days of intravenous remdesivir [7]. A total of 4241 patients were included in this meta-analysis. Of these, 2115 patients were randomly assigned to receive anti-viral agents as the intervention, and 2126 patients received a placebo as the controls.

**Figure 2:**
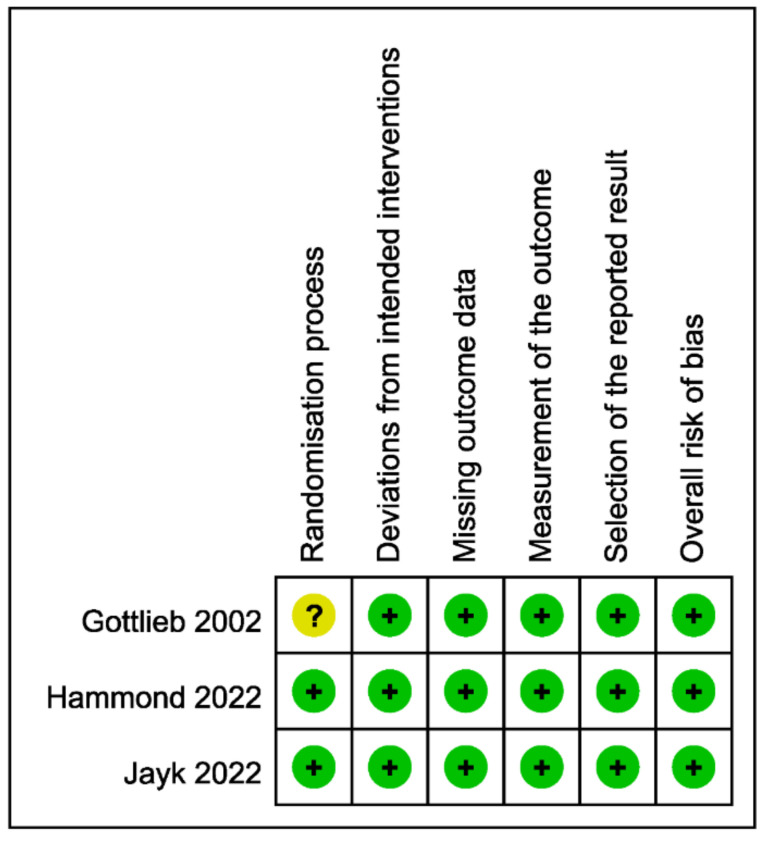
Risk of bias assessment [5,6,7].

Figure 2 shows the results of the risk of bias assessment; all three studies were assessed as having a low risk of bias, although one study [7] had some concerns related to the randomization process.

### 3.2. Primary Outcome

**Figure 3:**
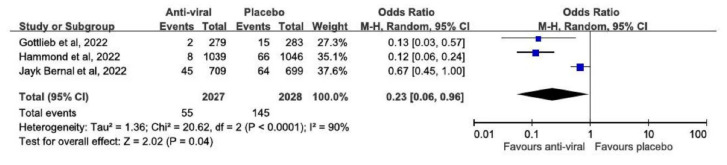
Meta-analysis of the associations of anti-viral agents versus placebo with the risk of COVID-19-related hospitalization or death [5,6,7].

Overall, anti-viral agents were associated with a significantly lower risk of COVID-19-related hospitalization or death compared with the placebo (OR, 0.23; 95% CI: 0.06–0.96; *p* = 0.04), but high heterogeneity was detected (*I*^2^ = 90%; *p* < 0.0001; Figure 3). This finding remained unchanged when applying leave-one-out sensitivity tests. In addition, significantly lower all-cause mortality was observed for the study group receiving anti-viral agents compared with the control group receiving a placebo (OR, 0.04; 95% CI: 0.01–0.33; *p* = 0.002), and no heterogeneity was detected (*I*^2^ = 0%; *p* = 0.90).

Finally, two RCTs reported the rate of all-cause hospitalization or death, and an analysis of these two studies [6,7] revealed that anti-viral agents were associated with a significantly lower risk of all-cause hospitalization or death compared with the placebo (OR, 0.59; 95% CI: 0.41–0.84; *p* = 0.003).

### 3.3. Safety Outcomes

**Figure 4:**
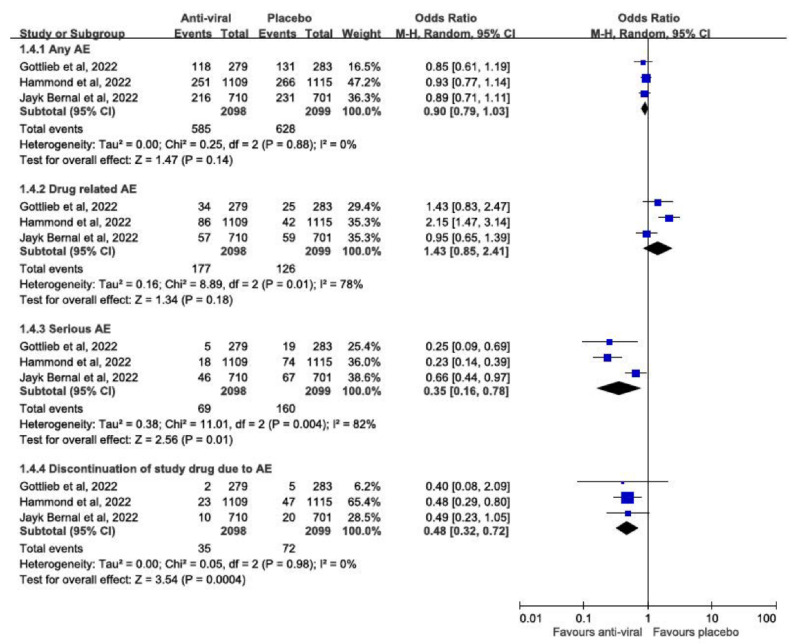
Meta-analysis of the risk of AEs between anti-viral agents and placebo [5,6,7].

Compared with a placebo, anti-viral agents were associated with a similar risk of any AEs (OR, 0.90; 95% CI: 0.79–1.03; *p* = 0.14) and drug-related AEs (OR, 1.43; 95% CI: 0.85–2.41; *p* = 0.18). Additionally, anti-viral agents were associated with significantly lower risks of serious AEs (OR, 0.35; 95% CI: 0.16–0.78; *p* = 0.01) and study drug discontinuation due to AEs (RR, 0.48; 95% CI: 0.32–0.72; *p* = 0.004) (Figure 4).

### 3.4. Network Meta-Analysis

**Figure 5:**
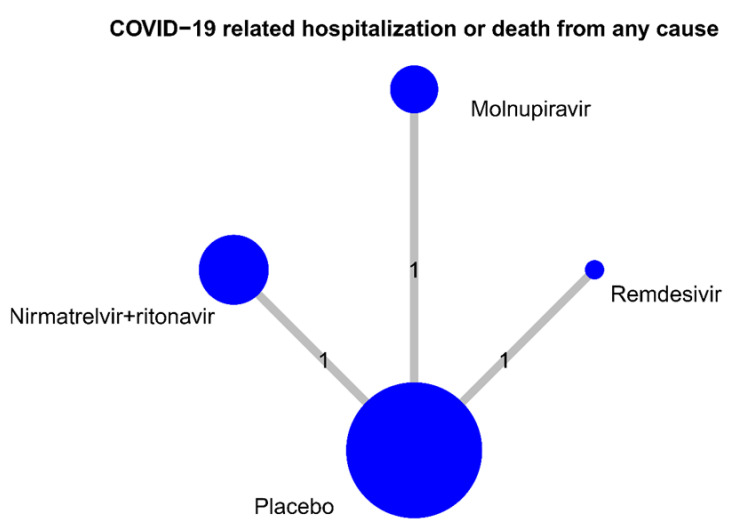
Number of studies for pairwise of antiviral drugs. The size of circle is proportional to sample size of each drug.

**Figure 6:**
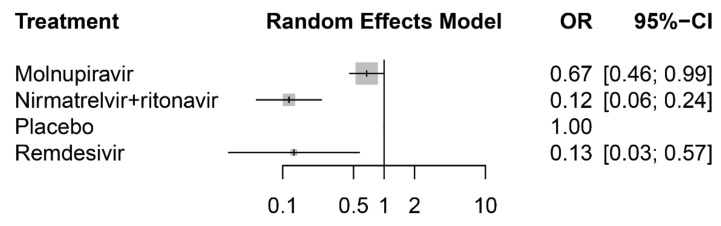
Forest plot of the network meta-analysis for COVID-19-related hospitalization or death from any cause.

The network meta-analysis included all three RCTs, which provided results for the risk of COVID-19-related hospitalization or death. Each of them had a direct comparison between a placebo and anti-viral agents: either oral nirmatrelvir plus ritonavir [5], oral molnupiravir [6] or intravenous remdesivir [7] (Figure 5). Compared with the placebo, patients receiving nirmatrelvir plus ritonavir had the lowest risk of hospitalization or death (OR, 0.12; 95% CI: 0.06–0.24), followed by remdesivir (OR, 0.13; 95% CI: 0.03–0.57) and then molnupiravir (OR, 0.67; 95% CI: 0.46–0.99) (Figure 6). Further pairwise comparisons in the network meta-analysis between antiviral drugs for COVID-19 showed that nirmatrelvir plus ritonavir and remdesivir were associated with a significantly lower risk of hospitalization or death compared with molnupiravir and the placebo (Table 2). Finally, the rank probability for each treatment, calculated using the P-score, is presented in Table 3. The results showed that nirmatrelvir plus ritonavir was the best anti-viral treatment, followed by remdesivir and then molnupiravir.

## 4. Discussion

The present study investigated the clinical efficacy and safety of anti-viral agents for non-hospitalized patients with COVID-19. Our results indicated that anti-viral agents, including nirmatrelvir plus ritonavir, molnupiravir and remdesivir, were effective for the prevention of disease progression in high-risk, non-hospitalized patients with COVID-19, as supported by the following evidence. First, a significantly lower risk of COVID-19-related hospitalization or death was observed in the study group receiving anti-viral agents compared with the control group receiving a placebo. Second, no mortality was reported in the study group, but 21 all-cause deaths were observed in the control group, which is a significant difference. Third, the study group also had a lower risk of all-cause hospitalization compared with the control group. The above findings were all consistent with two real-world studies that included both unvaccinated and vaccinated patients [16,17].

In Israel, a population-based study of 180,351 patients showed that nirmatrelvir plus ritonavir was associated with a significant decrease in the risk of severe COVID-19 or mortality (adjusted HR 0.54; 95% CI, 0.39–0.75) [16]. In Hong Kong, a retrospective cohort of 1,072,004 non-hospitalized COVID-19 patients showed that molnupiravir use was associated with a lower risk of mortality (HR, 0.61; 95% CI, 0.46–0.82) and in-hospital composite outcomes (HR, 0.64; 95% CI, 0.50–0.83) compared with non-molnupiravir users [17]. In addition, nirmatrelvir/ritonavir use was associated with a lower risk of mortality (HR, 0.25; 95% CI, 0.13–0.47), hospitalization (HR, 0.69; 95% CI, 0.60–0.79, *p* < 0.001), and in-hospital outcomes (HR, 0·47; 95% CI, 0.31–0.71) compared with non-nirmatrelvir/ritonavir users [17]. In addition, our network meta-analysis further indicated that nirmatrelvir plus ritonavir and remdesivir were better than molnupiravir for the prevention of COVID-19-related hospitalization or death. Although we did not evaluate their virological efficacy, both anti-viral agents (nirmatrelvir plus ritonavir and molnupiravir) showed a greater reduction in mean viral loads from baseline to day 5 compared with the placebo. Therefore, for non-hospitalized patients with COVID-19 who are at high risk of disease progression, these three anti-viral agents (nirmatrelvir plus ritonavir, molnupiravir and remdesivir) should be recommended. Among them, oral nirmatrelvir plus ritonavir and intravenous remdesivir may be better choices than molnupiravir.

All these findings were consistent with previous meta-analyses [18] regarding the efficacy of anti-viral therapies for outpatients with COVID-19. In Wen et al.’s study [18], the overall OR for mortality or hospitalization was 0.33 (95% CI, 0.22–0.49) for COVID-19 patients in the study group, in contrast with the placebo group. However, it should be noted that this meta-analysis included eight studies that assessed three anti-viral agents, including nirmatrelvir plus ritonavir, molnupiravir and fluvoxamine, and many of the included studies from that meta-analysis were not peer reviewed; in addition, fluvoxamine is not recommended in the current guidance [4,12,13]. Notably, several other meta-analyses have reported the usefulness of other anti-viral agents, such as lopinavir/ritonavir, favipiravir, umifenovir, sofosbuvir/daclatasvir, sofosbuvir/ledipasvir and sofosbuvir/velpatasvir [19,20,21,22]; however, the roles of these agents remain unclear, and most of the treatment guidelines [4,12,13] did not recommend their use. In contrast, the present study included three double-blind RCTs, and all three anti-viral agents, including nirmatrelvir plus ritonavir, molnupiravir and remdesivir, are recommended in the current treatment guidelines [4,12,13]. Therefore, our study provides more robust evidence and up-to-date information on this ongoing issue.

Finally, this study assessed the safety of three anti-viral agents for the treatment of outpatients with COVID-19. This meta-analysis did not find that these agents increased the risk of any AEs or drug-related AEs compared with the placebo. These findings were consistent with previous phase 2 studies of molnupiravir [23]. Moreover, a lower risk of serious AEs and discontinuation of the drug due to AEs was observed in the study groups compared with the control group. Therefore, our findings suggest that these three guidelines recommended anti-viral agents are a safe therapeutic option.

It should be noted that this study had several limitations. First, the number of included studies was limited because only RCTs were included in this study, and we excluded observational or cohort studies. However, RCTs can provide more stronger evidence than real-world studies. Therefore, the level of the evidence in the present meta-analysis based on RCTs should be considered robust. Second, all three RCTs assessed unvaccinated COVID-19 patients who were at an increased risk of severe illness, so we do not know their effects in vaccinated patients or those who are at a low risk of disease progression. Some studies, such as EPIC-SR (NCT05011513) [24] and several RCTs in India (Table S2), are ongoing, and further investigations are warranted. However, about one-third of population in the world has not received any vaccination till now [25]. This study can provide updated information regarding the management of this population after they contract COVID-19.

## 5. Conclusions

In conclusion, for non-hospitalized patients with COVID-19 who are at high risk of disease progression, the currently recommended anti-viral agents, nirmatrelvir plus ritonavir, molnupiravir and remdesivir, should continue to be recommended for the prevention of disease progression. Among them, oral nirmatrelvir plus ritonavir and intravenous remdesivir seem to be more effective than molnupiravir according to this network meta-analysis. Finally, the three investigated anti-viral agents were shown to be as tolerable as the placebo in this clinical setting.

## Figures and Tables

**Table 1:** Characteristics of included studies.

Study	Design	Period	Site	Subjects	Timing	Study Drug	Comparator	No of Patients under Randomization		Primary Outcome
								Study Group	Control Group	
Gottlieb et al., 2022	Phase 3, double-blind, randomized, placebo-controlled trial	From 18 September 2020, through 8 April 2021	64 sites in the United States, Spain, Denmark, and the United Kingdom	Nonhospitalized, unvaccinated patients with COVID-19 who had at least one risk factor for disease progression	within 7 days after the onset of signs or symptoms	intravenous remdesivir (200 mg on day 1 and 100 mg on days 2 and 3)	Placebo	279	283	COVID-19–related hospitalization or death from any cause by day 28
Hammond et al., 2022	Phase 2–3, double-blind, randomized, placebo-controlled trial	Between 16 July and 9 December 2021	343 sites in multination	Nonhospitalized, unvaccinated adults with COVID-19 who were at high risk for progression to severe disease	within 5 days after the onset of signs or symptoms	300 mg of nirmatrelvir plus 100 mg of ritonavir, every 12 h for 5 days	Placebo	1120	1126	COVID-19–related hospitalization or death from any cause through day 28
Jayk Bernal et al., 2022	Phase 3, double-blind, randomized, placebo-controlled trial	Between 6 May 2021 and 4 November 2021	107 sites in 20 countries	Nonhospitalized, unvaccinated adults with mild-to-moderate, laboratory-confirmed COVID-19 and at least one risk factor for severe COVID-19 illness	within 5 days after the onset of signs or symptoms	molnupiravir (800 mg) orally twice daily for 5 days	Placebo	716	717	hospitalization or death through day 29

**Table 2:** Results of the pairwise comparisons in the network meta-analysis between antiviral agents for COVID-19.

Antiviral Agents *	Nirmatrelvir Plus Ritonavir	Remdesivir	Molnupiravir	Placebo
Nirmatrelvir plus ritonavir		0.89 (0.17–4.69)	0.17 (0.07–0.39)	0.12 (0.06–0.24)
Remdesivir	1.12 (0.21–5.88)		0.19 (0.04–0.89)	0.13 (0.03–0.57))
Molnupiravir	5.85 (2.54–13.46)	5.22 (1.13–24.22)		0.67 (0.46–0.99)
Placebo	8.68 (4.15–18.17)	7.75 (1.76–34.22)	1.48 (1.01–2.18)	

* Odds ratio and 95% confidence interval were presented with drugs on the column as the reference.

**Table 3:** Rank probabilities for treatment by P-score.

Antiviral Agents	*p*-Score *
Nirmatrelvir + ritonavir	0.8510
Remdesivir	0.8087
Molnupiravir	0.3317
Placebo	0.0086

* Higher probability indicates better treatment for COVID-19.

## Data Availability

Not applicable.

## Supplementary Materials

The following supporting information can be downloaded at: https://www.mdpi.com/article/10.3390/v14081706/s1, Table S1: Search strategy; Table S2: Registered clinical trials to investigate the efficacy of anti-viral agents for COVID-19 in non-hospitalized adult patients, not yet published.
