# Supplementary material for: The Clinical Efficacy and Safety of Anti-Viral Agents for Non-Hospitalized Patients with COVID-19: A Systematic Review and Network Meta-Analysis of Randomized Controlled Trials
Source: Viruses. 2022 Aug 2;14(8):1706. doi: 10.3390/v14081706 (PMC9415971; doi:10.3390/v14081706)
Supplement: Supplementary file 1 [file viruses-14-01706-s001.zip › Table S1.pdf]

Table S1. Search strategy

Search strategy in Pubmed (Search date: 2022/05/28)

PICO	Search	Query	Items found	Time
	28	#22 AND #27	346	10:10:07
	27	#23 OR #24 OR #25 OR #26	1,571,527	10:09:55
	26	random*	1,565,640	10:09:50
	25	RCT	30,622	10:09:46
	24	Randomized Controlled Trial*	844,127	10:09:41
	23	Randomized Controlled Trial (MeSH)	756,831	10:09:35
**PI**	22	#10 AND #21	2,920	10:09:23
	21	#11 OR #12 OR #13 OR #14 OR #15 OR #16 OR #17 OR #18 OR #19 OR #20	3,031	10:09:14
	20	'PF-07321332'	121	10:07:06
	19	Paxlovid	69	10:07:00
	18	nirmatrelvir	109	10:06:49
	17	'EIDD-2801'	229	10:06:46
	16	'MK-4482'	213	10:06:40
	15	Lagevrio	211	10:06:34
	14	Molnupiravir	211	10:06:28
	13	'GS-5734'	2,806	10:06:18
	12	Veklury	2,802	10:06:09
**I**	11	Remdesivir	2,802	10:05:47
	10	#1 OR #2 OR #3 OR #4 OR #9	282,880	10:05:39
	9	#5 AND (#6 OR #7 OR #8)	4,908	10:05:25
	8	infection*	2,264,746	10:05:16
	7	viral	963,005	10:05:11
	6	virus	1,340,862	10:05:07
	5	corona	16,788	10:04:59
	4	sars-cov-2	163,296	10:04:51
	3	covid*	253,987	10:04:45
	2	coronavir*	133,194	10:04:36
**P**	1	Coronavirus Infections [MeSH term]	178,679	10:04:15

Search strategy in Embase (Search date: 2022/05/28)

PICO	No.	Query	Results
	#28	#23 AND #27	919
	#27	#24 OR #25 OR #26	2053410
	#26	random*	2040711
	#25	rct	52944
	#24	'randomized controlled trial'/exp [Emtree term]	713457
PI	#23	#11 AND #22	8065
	#22	#12 OR #13 OR #14 OR #15 OR #16 OR #17 OR #18 OR #19 OR #20 OR #21	8242
	#21	‘pf-07321332’	58
	#20	paxlovid	101
	#19	nirmatrelvir	178
	#18	‘eidd-2801’	89
	#17	‘mk-4482’	37
	#16	lagevrio	11
	#15	molnupiravir	343
	#14	‘gs-5734’	266
	#13	veklury	80
**I**	#12	remdesivir	7987
	#11	#1 OR #2 OR #3 OR #4 OR #5 OR #10	323001
	#10	#6 AND (#7 OR #8 OR #9)	6157
	#9	infection*	3088022
	#8	viral	637168
	#7	virus	1695483
	#6	corona	23526
	#5	'sars-cov-2'	99635
	#4	covid*	259053
	#3	coronavir*	274294
	#2	'coronavirus disease 2019'/exp [emtree term]	219391
**P**	#1	'coronavirinae'/exp [emtree term]	95128

Search strategy in **Web of Science Core Collection** (Search date: 2022/05/28)

PICO	#	Query	Results
	#26	#21 AND #25	255
	#25	#22 OR #23 OR #24	1,987,769
	#24	ALL=(random*)	1,980,262
	#23	ALL=(rct)	32,398
	#22	ALL=('randomized controlled trial')	475,803
**PI**	#21	#9 AND #20	2,501
	#20	#10 OR #11 OR #12 OR #13 OR #14 OR #15 OR #16 OR #17 OR #18 OR #19	2,652
	#19	ALL=('PF-07321332')	34
	#18	ALL=(Paxlovid)	31
	#17	ALL=(nirmatrelvir)	40
	#16	ALL=('EIDD-2801')	48
	#15	ALL=('MK-4482')	24
	#14	ALL=(Lagevrio)	5
	#13	ALL=(Molnupiravir)	138
	#12	ALL=('GS-5734')	206
	#11	ALL=(Veklury)	22
**I**	#10	ALL=(Remdesivir)	2,446
	#9	#1 OR #2 OR #3 OR #8	321,626
	#8	#4 AND (#5 OR #6 OR #7)	6,031
	#7	ALL=(infection*)	1,863,349
	#6	ALL=(viral)	451,586
	#5	ALL=(virus)	1,027,485
	#4	ALL=(corona)	59,971
	#3	ALL=('sars-cov-2')	81,105
	#2	ALL=(covid*)	282,252
**P**	#1	ALL=(coronavir*)	115,361

Search strategy in Cochrane Library (Search date: 2022/05/28)

PICO	Search	Query
P	#1	Coronavirus Infections [MeSH term]
	#2	coronavir*
	#3	covid*
	#4	sars-cov-2
	#5	corona
	#6	virus
	#7	viral
	#8	infection*
	#9	#5 AND (#6 OR #7 OR #8)
	#10	#1 OR #2 OR #3 OR #4 OR #9
I	#11	Remdesivir
	#12	Veklury
	#13	'GS-5734'
	#14	Molnupiravir
	#15	Lagevrio
	#16	'MK-4482'
	#17	'EIDD-2801'
	#18	nirmatrelvir
	#19	Paxlovid
	#20	'PF-07321332'
	#21	#11 OR #12 OR #13 OR #14 OR #15 OR #16 OR #17 OR #18 OR #19 OR #20
	#22	#10 AND #21

Cochrane Database of Systematic Reviews (CDSR): 16

Cochrane Central Register of Controlled Trials (CENTRAL): 334

Search strategy in **clinicaltrials.gov** (Search date: 2022/05/28)

Keyword: Remdesivir OR Veklury OR 'GS-5734' OR Molnupiravir OR Lagevrio OR 'MK-4482' OR 'EIDD-2801' OR nirmatrelvir OR Paxlovid OR 'PF-07321332' | Covid19

Result: 155 trials

Search strategy in **WHO International Clinical Trials Registry Platform**

(Search date: 2022/05/28)

Keyword: Remdesivir OR Veklury OR 'GS-5734' OR Molnupiravir OR Lagevrio OR 'MK-4482' OR 'EIDD-2801' OR nirmatrelvir OR Paxlovid OR 'PF-07321332'

Result: 157 trials
