# Supplementary material for: The Clinical Efficacy and Safety of Anti-Viral Agents for Non-Hospitalized Patients with COVID-19: A Systematic Review and Network Meta-Analysis of Randomized Controlled Trials
Source: Viruses. 2022 Aug 2;14(8):1706. doi: 10.3390/v14081706 (PMC9415971; doi:10.3390/v14081706)
Supplement: Supplementary file 1 [file viruses-14-01706-s001.zip › Table S2.pdf]

Table S2. Registered clinical trials to investigate the efficacy of anti-viral agents for COVID-19 in non-hospitalized adult patients, not yet published.

Registration Number	Country	Local version of which trial	Study Design	Subjects	Timing	Study drug	Comparator	Primary outcome	Status
NCT05011513 EUCTR2021-002857-28	Argentina, Brazil, Bulgaria, Colombia, Czechia, Hungary, Japan, Republic of Korea, Malaysia, Mexico, Poland, Puerto Rico, Russian Federation, South Africa, Spain, Taiwan, Thailand, Turkey, Ukraine, United States	EPIC-SR	Phase 2-3, quadruple-blind, randomized, placebo-controlled trial	Non-hospitalized patients with Covid-19	within 5 days after the onset of signs or symptoms	300 mg of nirmatrelvir plus 100 mg of ritonavir, every 12 hours for 5 days	Placebo	Time to sustained alleviation of all targeted COVID-19 signs/symptoms [through Day 28]	Recruiting
CTRI/2021/05/033693	India	CBCC/2021/008	Phase 3, open label, randomized controlled trial	Non-hospitalized patients with Covid-19	Not mentioned	molnupiravir (800 mg) orally twice daily for 5 days	Standard of care	Rate of hospitalization up to Day 14	Open to Recruitment
CTRI/2021/05/033739	India	HCR/III/MOLCOV/04/2021-01	Phase 3, open label, randomized controlled trial	Non-hospitalized patients with Covid-19	Not mentioned	molnupiravir (800 mg) orally twice daily for 5 days	Standard of care	Rate of hospitalization up to Day 14	Completed
CTRI/2021/05/033904	India	MOLN/MSN/P3-M1/2021	Phase 3, open label, randomized controlled trial	Non-hospitalized patients with Covid-19	within 7 days after the onset of signs or symptoms	molnupiravir (800 mg) orally twice daily for 5 days	Standard of care	Rate of hospitalization up to Day 14	Completed

CTRI/2021/06/033938	India	DRL-MOL-002	Phase 3, open label, randomized controlled trial	Non-hospitalized patients with Covid-19	within 5 days after the onset of signs or symptoms	molnupiravir (800 mg) orally twice daily for 5 days	Standard of care	Rate of hospitalization up to Day 14	Open to Recruitment
CTRI/2021/06/033992	India	OPT-MOL-001	Phase 3, open label, randomized controlled trial	Non-hospitalized patients with Covid-19	within 5 days after the onset of signs or symptoms	molnupiravir (800 mg) orally twice daily for 5 days	Standard of care	Rate of hospitalization up to Day 14	Closed to Recruitment
CTRI/2021/06/034015	India	NV-05-1154-2021	Phase 3, open label, randomized controlled trial	Non-hospitalized patients with mild COVID-19 with a score of 2 or 3 on the 10-point ordinal scale of clinical status	within 3-5 days after the onset of signs or symptoms	molnupiravir (800 mg) orally twice daily for 5 days	Standard of care	Rate of hospitalization up to Day 14	Open to Recruitment
CTRI/2021/06/034130	India	BDR/CT/004/MOL/2021	Phase 3, open label, randomized controlled trial	Non-hospitalized patients with Covid-19	within 5 days after the onset of signs or symptoms	molnupiravir (800 mg) orally twice daily for 5 days	Standard of care	Rate of hospitalization up to Day 14	Completed
CTRI/2021/07/034588	India	CR216-21	Phase 3, open label, randomized controlled trial	Non-hospitalized patients with Covid-19 who had at least one risk factor for disease progression	within 5 days after the onset of signs or symptoms	molnupiravir (800 mg) orally twice daily for 5 days	Standard of care	Rate of hospitalization from randomization up to Day 14.	Completed
